# The effect of context and reason on the neural correlates of intentions

**DOI:** 10.1016/j.heliyon.2023.e17231

**Published:** 2023-06-15

**Authors:** Sebo Uithol, Kai Görgen, Doris Pischedda, Ivan Toni, John-Dylan Haynes

**Affiliations:** a Cognitive Psychology Unit, Institute of Psychology & Leiden Institute for Brain and Cognition, Leiden University, Leiden, the Netherlands; b Bernstein Center for Computational Neuroscience, Charité – Universitätsmedizin Berlin, Corporate Member of Freie Universität Berlin and Humboldt-Universität zu Berlin, Berlin, Germany; c Berlin Center for Advanced Neuroimaging, Charité – Universitätsmedizin Berlin, Corporate Member of Freie Universität Berlin and Humboldt-Universität zu Berlin, Berlin, Germany; d Department of Brain and Behavioral Sciences, University of Pavia, Pavia, Italy; e Donders Institute for Brain, Cognition and Behaviour, Radboud University Nijmegen, the Netherlands; f Science of Intelligence, Research Cluster of Excellence, Berlin, Germany; g Humboldt-Universität zu Berlin, Berlin School of Mind and Brain and Institute of Psychology, Berlin, Germany; h Technische Universität Dresden; SFB 940 Volition and Cognitive Control, Dresden, Germany

**Keywords:** Intentional action, Action selection, Context-dependence, fMRI, MVPA, Embodiment

## Abstract

Many studies have identified networks in parietal and prefrontal cortex that are involved in intentional action. Yet, our understanding of the way these networks are involved in intentions is still very limited. In this study, we investigate two characteristics of these processes: context- and reason-dependence of the neural states associated with intentions. We ask whether these states depend on the context a person is in and the reasons they have for choosing an action. We used a combination of functional magnetic resonance imaging (fMRI) and multivariate decoding to directly assess the context- and reason-dependency of the neural states underlying intentions. We show that action intentions can be decoded from fMRI data based on a classifier trained in the same context and with the same reason, in line with previous decoding studies. Furthermore, we found that intentions can be decoded across different reasons for choosing an action. However, decoding across different contexts was not successful. We found anecdotal to moderate evidence against context-invariant information in all regions of interest and for all conditions but one. These results suggest that the neural states associated with intentions are modulated by the context of the action.

## 1 Introduction

Intentions are believed to operate at the interface of thought and action [1], and therefore have to translate cognitive states into detailed motor coordination [2]. They are assumed to be the end state of a decision process, and to be the primary cause of the subsequent action [3]. In this role, intentions are thought to consist of an action plan, and the decision to execute this plan [1]. Due to this assumed central role intentions play in our actions, they have a rich history of scientific investigation, going back to at least William James [4] in psychology, and Kornhuber & Deecke [5] in physiology.

Even though the (folk) psychological notion of “intention” might be taken to imply a homogeneous or unitary process across different conditions, neuroscientific evidence suggests that multiple brain regions are involved in action intentions [[6], [7], [8], [9], [10]]. For example, human stimulation studies show that the *urge* to move can be distinguished from the *desire* to move [11]. While urges (the feeling of wanting to make a specific movement) are evoked when the medial prefrontal cortex—specifically the supplementary motor area [12]—is stimulated, a desire to make a movement (the feeling of wanting to make an unspecified movement) is evoked upon stimulation of the inferior parietal lobule [13].

In order to accommodate the multitude of brain areas involved in voluntary action and the various roles intentions are thought to play, Jahanshahi [14] suggested that intentions consist of multiple components, including a “what to do” component, a decision “when to act”, and an inhibitory process. A similar decomposition can be found in Brass and Haggard's [15] “what”, “when”, and “whether” model of intentional action. Using fMRI this model was subsequently refined, but also challenged. For instance pre-supplemental motor area (pre-SMA) and anterior cingulate cortex (ACC) were found to contribute to all three components of the “what, when and whether model” [16,17].

These findings and models explicate the stages and structures involved in intentional action. The ‘what component’ is construed to represent the content of an intention [18,19], and is associated with activity in the frontomedial cortex, including, the rostral cingulate cortex, the supplemental motor area and the pre-supplemental motor area. A hierarchical cluster analysis points more specifically to the right middle cingulum, the right middle frontal gyrus, the right supramarginal gyrus and the left inferior frontal gyrus and pars triangularis [17].

Yet, what exactly is contained in the ‘what’ component of intentions is beyond the scope of most empirical studies. A typical way of assessing the neural correlates of the what component is to contrast an endogenously performed action with a cued action. This contrast does inform us about the difference between cued and self-selected actions, but does not explain what the mechanisms of voluntary action are.

In everyday language use, and often in philosophy of action, an intention is generally framed as a state that is abstracted away from the immediate context of the intended action, and the reasons one can have for acting [20]. Neuroscience is often not explicit about how much of these processes are interpreted as part of (the what component of) an intention, or rather part of the processes leading up to the intention. Some work was done under the assumption that action decisions and action planning processes cannot be separated [[21], [22], [23]] and hence action intentions are context-dependent. Others have questioned this view [but see 24,25. Along similar lines, different reasons for performing an action have been shown to result in different kinematics [to the extent that they can be picked up by a human observer: [[26], [27], [28]], suggesting that different reasons may also be responsible for differences in the processes underlying intentional actions. Also in action observation, context is reported to modulate neural response in the observer [29].

In everyday life, we virtually never form intentions about abstract stimuli or objects. Almost always the intentions we form are about actions in a meaningful context, and for specific reasons. These factors are commonly not investigated in cognitive studies on intentions. In this exploratory study, we directly test the reason- and context-dependency of intention representations. We investigated the outcomes of action decisions, which we in the following refer to as “intentions”. Specifically, we investigate whether the same intention made for different reasons or in different contexts is accompanied by invariant neural patterns, or whether the patterns are context- or reason-dependent. To this purpose, we use multivariate pattern analyses (MVPA) of functional magnetic resonance imaging (fMRI) data [[30], [31], [32], [33], [34], [35]].

In this study, participants formed action intentions based on specific reasons and in specific contexts. Given a certain reason, only one of the actions was reasonable. However, participants were not explicitly instructed by us to make a particular choice. We chose to use such semi-free intentions for two reasons: First, in real life we virtually never form intentions that are completely free from reasons to act. Rather, we form intentions because we want to achieve a certain goal, say to go to the office. So, the actions we perform in the morning are performed *because* we want to go to the office showered and dressed.^1^ These actions are performed in a meaningful context (e.g., bathroom, bedroom). Second, this setup allows us to check that participants perform the task (by assessing their responses) and to balance the number of trials per condition.

## 2 Methods

### 2.1 Participants

Thirty participants took part in this study. Four of these were excluded from the neuroimaging analyses due to low performance (see 3.1), leaving N = 26 (19 female) for neuroimaging analysis. Participants were 18 to 37 years old (mean age 26.7 years). All participants had normal or corrected-to-normal vision and were right-handed according to the Edinburgh handedness assessment [37]. Participants had no history of neurological or psychiatric disorders and gave written informed consent. All participants mastered the German language at a native level, and received 20€ for participation. The study was approved by the local ethics committee (Ethikkommission Lebenswissenshaftliche Facultät, number 2016-07).

### 2.2 Experimental setup

**Fig. 1:**
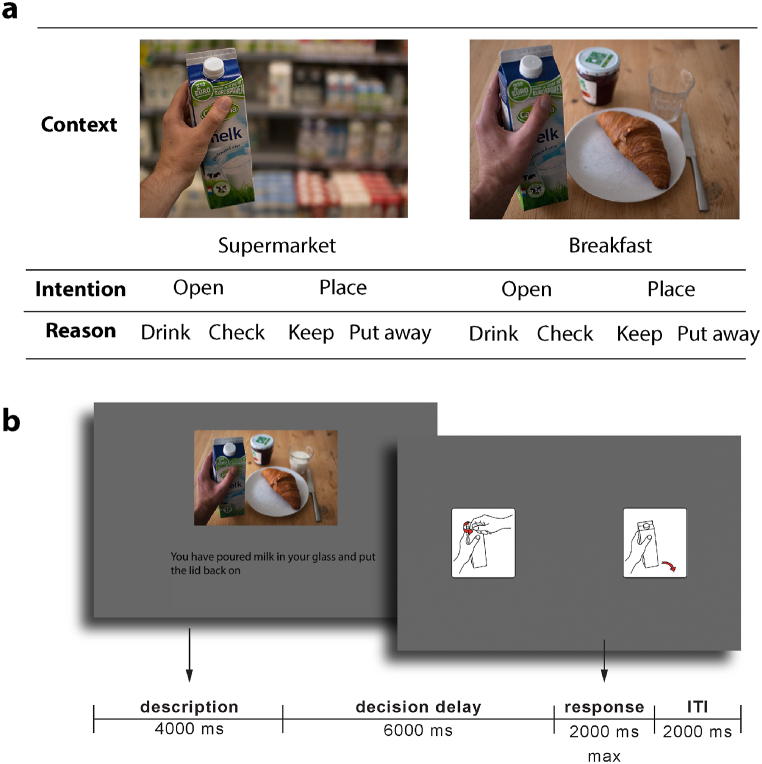
Experimental design. Panel **a**: schematic overview of the eight experimental conditions. The photos here are examples; various photos were used. Panel **b**: Graphical representation of one trial. The originally German sentence has been translated to English. During the delay and the intertrial interval (ITI) an empty grey screen was presented.

Stimuli were presented using PsychoPy version 1.83.03 [38]. They were projected onto a screen at the back of the scanner that was visible through a mirror mounted on the MR head coil. Each trial (Fig. 1) started with an image depicting a contextual setting (either a breakfast or a supermarket context), presented at the centre of the screen (factor “context”). Beneath the picture a one-sentence explanation of a situation was presented in German (factor “reason”). For example, “You have poured milk in your glass and you've put the lid back on”, which suggests placing the box (action) in order to do away with it (reason) in a breakfast context. Participants were instructed to imagine themselves in this situation and decide what would be the appropriate action in that situation. The combination of picture and sentence was presented for 4000 ms. This combination made only one of the intentions appropriate. Trials in which the participant failed to provide the answer that was suggested by this combination were considered incorrect and discarded from further analysis (Please see supplemental material for an overview of the trial with the correct responses). This screen was followed by a 6000 ms decision delay with an empty grey screen. After that, the response screen was presented, which always contained the two options “open” and “place”. On half of the trials these options were presented as words, on the other half of the trials as pictograms, to prevent participants from anticipating specific visual input. The two possible answers were displayed each on different sides of the screen, and participants indicated the correct answer by pressing a button with either their left or right index finger. The side on which each answer appeared was randomised to prevent a conflation between choice and motor preparation. Participants were asked to respond within 2000 ms. Responses that were made after 2000 ms were considered invalid. The response was followed by a 2000 ms intertrial interval (ITI) in which participants could prepare for the next trial. Since the ITI started as soon as the participant responded (i.e., not waiting for the full 2000 ms answering time to finish) a natural jitter occurred. Responses and response times were recorded.

There were two contexts (supermarket vs. breakfast), two action options (open vs. place) and two reasons for choosing one action or the other, resulting in a 2 × 2 x 2 design. As the reasons for opening were necessarily different from the reasons for placing (in order to provide ecologically plausible reasons), this factor was nested. Each of the eight conditions was repeated four times within one run. Participants performed five runs (separated by short breaks). This means that each condition was repeated 20 times, resulting in a total of 160 trials. Participants received instructions and a short training session prior to the experiment. During the training session, participants performed five trials, randomly selected from the experiment trials. The total duration of the experiment, without setting up and training, was approximately 40 min.

Trial order was randomised, and the experimental design was assessed using the Same Analysis Approach [39] to test for unintended regularities in the design (e.g. trial order, imbalances, design-analysis-interactions) or behavioural differences (error rates, reaction times) that could bias the machine-learning classifier (see below). No such unintended regularities were detected.

### 2.3 Image acquisition

A 3 T S Trio (Erlangen, Germany) scanner with a 12-channel head coil was used to collect functional magnetic resonance imaging data. In each run, 266 T^2^*-weighted echo-planar images (EPI) were acquired using a descending interleaved protocol (TR = 2030 ms, TE = 30 ms, flip angle 78°). Each volume consisted of 33 slices, separated by a gap of 0.75 mm. Matrix size was 64 × 64, and field of view (FOV) was 192 mm, which resulted in a voxel size of 3 × 3 × 3.75 mm. The first three images of each run were discarded, in order to allow the magnetic field to stabilise. Additionally, field distortion maps (TR = 400 ms, TE1 = 5.16 ms; TE2 = 7.65 ms; flip angle = 60 deg.) were collected for correcting the EPIs. A structural, T1-weighted image (1 mm isotropic voxels; TR = 1900 ms; TI = 900 ms; TE = 2.52 ms; flip angle = 9 deg.) was collected for anatomical localisation.

### 2.4 Data analysis

The EPI images were preprocessed using SPM12 (https://www.fil.ion.ucl.ac.uk/spm/software/spm12/). The images were realigned, unwarped and slice-time corrected. Next, a general linear model [GLM]; [40] was estimated with 12 regressors corresponding to the 8 conditions in the design (see Fig. 1, panel a) plus the stimulus pictures as nuisance regressors (in order to minimise a possible effect of visual information on the performance of the classifier in the subsequent analyses). Regressors were modelled as a box-car encompassing presentation of the description for the picture regressors (0–4000 ms from trial onset) and the decision delay period for the experimental conditions (4000-10,000 ms from trial onset, see Fig. 1) and convolved with the canonical hemodynamic response function. We also included 6 regressors with movement parameters as regressors of no interest. The condition-, voxel-, and run-wise parameter estimates of the resulting GLM were subsequently used as input for the multivariate analyses.

#### 2.4.1 Multivariate decoding

**Fig. 2:**
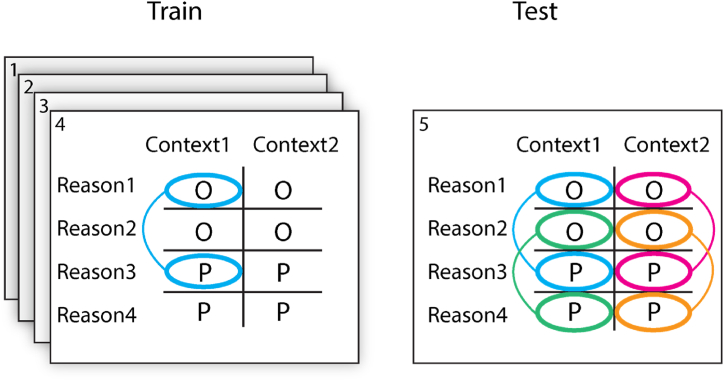
The training and testing procedure. For training and testing we employed a leave-one-run-out procedure. A classifier was trained to distinguish between the intention to open (O) and to place (P) on four of the five runs and subsequently tested on the left-out run in four different settings: 1) SameReasonSameContext (blue), 2) CrossReason (green), 3) CrossContext (magenta), and 4) CrossReasonCrossContext (orange). (For interpretation of the references to colour in this figure legend, the reader is referred to the Web version of this article.)

We performed multivariate pattern analysis (MVPA) using The Decoding Toolbox [TDT; 41]. A searchlight classifier [32,42] using libSVM [43] with a fixed linearisation parameter C1 was trained to classify the action (open vs. place) for one specific reason-context combination (See Fig. 2). The searchlights had a radius of 12 mm and were restricted by a whole-brain mask. This classifier was then subsequently tested on all four reason-context combinations in the remaining fifth run. For this, we employed 5-fold run-wise cross-validation [44] to estimate the generalisation performance of the classifier, by repeating this procedure with each run as left-out test data once, calculating the classification accuracy for each left-out run, and averaging the classification accuracies across runs. Results were corrected for multiple comparisons using a family-wise error correction at the cluster level (FWE_C_). The four training-test combinations were: (1) classifier trained and tested on the same reason and the same context (‘SameReasonSameContext’), (2) classifier trained and tested on the same context but on a different reason (‘CrossReason’), (3) classifier trained and tested on the same reason but in a different context (‘CrossContext’), and (4) classifier trained and tested on different reasons and different contexts, (‘CrossReasonCrossContext’, see Fig. 2). Training and testing was performed on unsmoothed images in individual space.

**Fig. 3:**
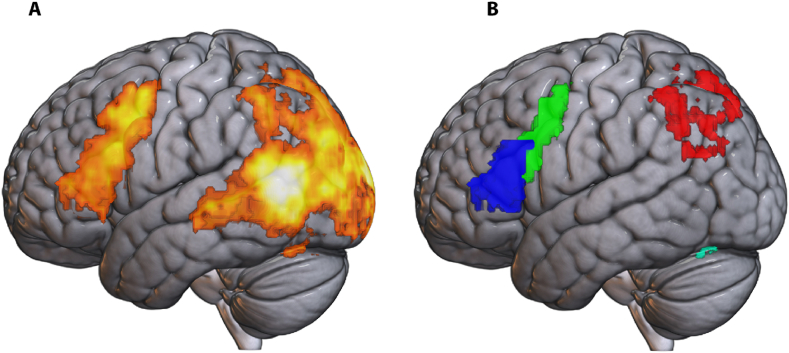
Regions of interest. **Panel A** shows searchlight decoding results with above-chance classification (*p* < 0.001, FWE_C_) in the SameReasonSameContext condition based on a second-level voxel-wise analysis including all subjects. **Panel B** shows the four participant-specific ROIs for participant 1 during the ROI analysis: prefrontal (blue); premotor (green); parietal (red); and cerebellum (cyan). To avoid circularity, the SameReasonSameContext, ROIs were created for each participant from the classification results of all other subjects (here: all except subject 1). (For interpretation of the references to colour in this figure legend, the reader is referred to the Web version of this article.)

In order to increase sensitivity, we restricted our crucial generalisation analyses to regions of interest (ROIs) that encoded intention-related information for the same context and the same reason (combination 1 in Fig. 2). To find these regions, we performed a decoding analysis in individual anatomical space, after which the decoding results were normalised to MNI space (3rd degree B-Spline interpolation) using the structural images and smoothed with a 2 × 2x2mm FWHM kernel), which allowed us to do a group-level analysis (second-level *t*-test for each voxel, *p* < 0.001, family-wise error correction at the cluster level (FWE_C_)). To avoid circularity in subsequent analyses in the estimate of the SameReasonSameContext condition [45,46], individual ROIs for each participant were created using a ‘leave-this-participant-out’ protocol (a leave-one-participant-out procedure in which, e.g., the ROIs of participant 1 were created using the decoding results of all subjects but participant 1, see also [7]. For this, a threshold of *p* < 0.001, FWE_C_, was used [47]. The ROIs covered a substantial part of the cortex, including visual, parietal, frontal, prefrontal and temporal regions (see Fig. 3, left panel). As we were not interested in visual decoding, and since the stimuli images were not completely matched in terms of luminosity, colour and detail, occipital cortex was excluded from further analysis, leaving parietal, premotor, prefrontal, and cerebellar areas. The continuous ROI was split into four functional-anatomical (fa)ROIs for each participant (using the Automated Anatomical Labeling library [48], which is based on the MNI anatomical labels). An example of these ROIs can be seen in Fig. 3, right panel; the exact boundaries of these ROIs varied slightly per participant. All ROIs were present on both hemispheres.

Next, we projected each ROI to the participant space of the left-out participant using the inverse of the transformation matrices that were used to normalise the images to MNI space. Subsequently, the classification accuracies were extracted from the whole-brain searchlight analysis for each of the ROIs. These accuracies were combined in another second-level analysis (again one-sample *t*-test). The group average classification in the four conditions (SameReasonSameContext, Different Reason, Different Context, and Different Reason Different Context) was compared in each of the four ROIs. See the supplemental material for a graphical overview of the analysis pipeline.

## 3 Results

### 3.1 Behavioural results

Only participants with two (out of four) or more correct trials on each of the eight conditions in each run were included in subsequent analyses. Four participants were excluded based on this criterion, leaving N = 26 participants for the fMRI analysis (see 2.1 above). Trials that contained responses that were either incorrect or that occurred after the instructed response window of 2000 ms were discarded. In total, 267 trials (6.4%) were discarded. On average, participants gave the expected answer in 93.6% of the trials (standard deviation 6.5%). Since there was a 6000 ms delay between stimulus presentation and answering screen, we assumed that no meaningful information can be drawn from the reaction times. This was confirmed by the outcome of a repeated-measure ANOVA, which showed no significant effect of condition on reaction time (*p* = 0.6).

### 3.2 Classification results

**Fig. 4:**
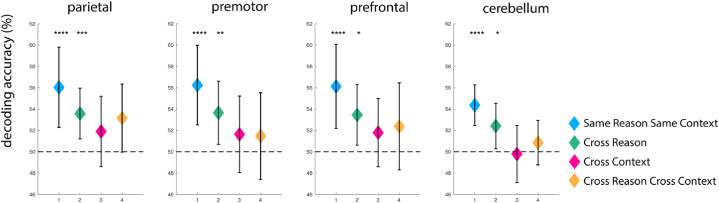
Results of the decoding analyses in each ROI. Asterisks denote values being significantly (*p* < 0.05, Bonferroni-corrected for multiple comparisons, *p* < 0.003 for each test) above-chance level (50%, horizontal dashed line). Error bars represent the 95% confidence interval (Bonferroni corrected for multiple comparison). Stars represent significance levels: ‘****’: *p* < 0.001; ‘***’: *p* < 0.005; ‘**’: *p* < 0.01; ‘*’: *p* < 0.05 (corrected).

The SameReasonSameContext condition showed significant information in all ROIs (mean classification accuracies: parietal: 56.0%; premotor: 56.2%; prefrontal: 56.2%; cerebellum: 54.3%; one-sided, one-sample *t*-test: *p* < 0.001 for all ROIs; the Bonferroni corrected significance threshold for multiple comparisons corresponding to α_bonf_ = 0.05 for four conditions and four ROIs is α_uncorr_ = 0.003; Cohen's d for the for conditions respectively: 0.9; 1.0; 0.9; 1.3; see Fig. 4). Please note that while the SameReasonSameContext condition was used to define the ROIs with a searchlight analysis, the ROI definition for each participant was created from the results of all other participants, and thus voxel selection and test-set classification performance were independent (i.e., “non-circular”). This allowed us to compare the results from different ROI conditions. Within each participant, the classification was performed using a leave-one-run-out cross-validation. Reason and context modulation were checked using repeated-measures ANOVA. This yielded a significant modulation for context in parietal cortex (p = 0.03), premotor cortex (p = 0.03), and cerebellum (p = 0.001). Reason was not significant in any of the four ROIs.

CrossContext decoding was not significant from chance level in any of the four ROIs (parietal: 51.9%; premotor: 51.6%; prefrontal: 51.8%; cerebellum: 49.8%, *p* > 0.05 for all ROIs, α = 0.003, α_bonf_ = 0.05; Cohen's d for the four conditions respectively: 0.3; 0.3; 0.3; −0.1), neither was CrossReasonCrossContext decoding (parietal: 53.2%, *p* = 0.003; premotor: 51.4%, *p* = 0.1; prefrontal: 52.3%, *p* = 0.05; cerebellum 50.1%, *p* > 0.1, α = 0.003, α_bonf_ = 0.05; Cohen's d for the four conditions respectively: 0.6; 0.2; 0.3; 0.6); see Fig. 4).

Next, we quantified the evidence for the absence of information in the neural data using Bayesian statistics by calculating the Bayes factors (BFs) for each condition using JASP [49]. The BFs were calculated using a Bayesian repeated-measures ANOVA over classification accuracies. Specifically, we calculated the BF_10_, which informs how more likely Hypothesis 1 (there being an effect) is than Hypothesis 0 (there being no effect) using a default prior (Cauchy, scale 0.707). Note that “10” in “BF_10_” indicates that it assesses Hypothesis 1 vs. Hypothesis 0, and not the base of the logarithm used below (which is the natural logarithm base *e*). Following Lee & Wagenmakers [50], we consider logarithms of the Bayes Factors |log_e_ (BF_10_)| between 0 and 1 to be anecdotal (grey in Table 1, Table 2); between 1 and 2 moderate (Table 1, Table 2); between 2 and 3.5 strong, between 3.5 and 4.5 very strong (not in our results), and larger than 4.5 as extreme. Negative values indicate evidence for the null hypothesis H0, that is, the absence of information suitable for classification, positive values indicate evidence for H1. The Bayesian repeated-measure ANOVA indicates moderate to extreme evidence for the presence of information about action in the CrossReason analysis in all ROIs, and anecdotal (one ROI) to moderate (three ROIs) evidence for H0 (that is, against the presence of information suitable for classification) for the CrossContext analysis (see Table 1).

**Table 1:** The natural logarithm of the Bayes Factors assessing H1 vs H0 (BF_10_) of the Bayesian repeated measures ANOVA for information on the action intentions vs. no information in the different ROIs. Negative values indicate evidence for the null hypothesis. Note: “10” in “BF_10_” indicates that it assesses Hypothesis 1 vs. 0; that is, it is not indicating the base of the logarithm that appears in the column titles, which is the natural logarithm (base *e*). Evidence level: |log_e_ (BF_10_)| 0–1: anecdotal; 1–2: moderate; 2–3.5: strong, 3.5–4.5: very strong (not in our results), >4.5: extreme.

	log_e_ (BF_10_) ANOVA CrossReason	log_e_ (BF_10_) ANOVA CrossContext
Parietal	1.9 (moderate H1)	−1.4 (moderate H0)
Premotor	−0.9 (anecdotal H0)	2.9 (strong H1)
Prefrontal	−1.1 (moderate H0)	1.0 (moderate H1)
Cerebellum	−1.4 (moderate H0)	7.3 (extreme H1)

**Table 2:** Logarithms of the Bayes Factors (BF_10_) for information on the action intentions vs. no information in the different ROIs. Negative values signify evidence for the null hypothesis that no information is present in the neural activity pattern. Notation, evidence level, and colour code are as in Table 1.

	log_e_ (BF_10_) T-Test SameReasonSameContext	log_e_ (BF_10_) T-Test CrossReason	log_e_ (BF_10_) T-Test CrossContext	log_e_ (BF_10_) T-Test CrossReasonCrossContext
Parietal	5.8 (extreme H1)	5.2 (extreme H1)	−0.3 (anecdotal H0)	1.8 (moderate H1)
Premotor	6.4 (extreme H1)	3.4 (strong H1)	−0.8 (anecdotal H0)	−1.1 (moderate H0)
Prefrontal	5.6 (extreme H1)	3.2 (strong H1)	−0.4 (anecdotal H0)	−0.8 (anecdotal H0)
Cerebellum	10.6 (extreme H1)	2.8 (strong H1)	−0.3 (anecdotal H0)	−0.9 (anecdotal H0)

Based on the significant CrossReason effect of the ANOVA, we performed separate Bayesian *t*-tests for each individual condition. Table 2 shows the logarithms of the Bayes Factors |log_e_ (BF_10_)| for all conditions in all ROIs. Again, negative values indicate evidence for H0 that no information suitable for classification is available for this condition in this ROI, H1 indicates evidence for the presence of information.

## 4 Discussion

In this paper, we measure relatively stable patterns of brain activation that can be correlated to the presence of an intentions in the participant. These patterns can be said to represent the attributed intention, although this may not necessarily imply the presence of a contentful neural representation [51,52]. We will use the term ‘neural state’ or ‘neural representation’ to refer to the patterns of brain activity identified using multivariate fMRI decoding techniques and that we, researchers, were able to correlate with the presence of an intention, without further assumption about the nature of these states.

We directly assessed the impact of changing context and changing reason on the voxel patterns accompanying intentions. We used MVPA on fMRI data to compare classification accuracy for same-context or same-reason conditions with the accuracy for cross-context and cross-reason conditions. When *context* changed between training and test data, the decoding accuracy dropped to chance level. Bayesian analyses showed in most cases moderate evidence for absence of context-independent information. Changing the *reason* for forming a certain intention did not have this effect. This suggests that context plays a crucial role in the neural states related to action intentions, in line with embodied approaches to cognition, that emphasise the ‘situatedness’ of cognition [53].

The notion of a context-invariant encoding of task information in a single brain region has been challenged before [see 54,55. In fact, it has long been known that different variables of tasks are encoded in different regions across cortex, supporting a distributed model of task encoding [8,[56], [57], [58]]. Furthermore, when preparing actions, information about ‘what’ will be performed and ‘when’ it will be performed is encoded in dissociable brain regions [10,17], in line with Brass and Haggard's [15] distinction of the ‘what’, ‘when’ and ‘whether’ subprocesses of intentions [8,9,59]. Our results can be interpreted as another extension of this heterogeneous neural implementation by showing that the ‘what’ component is at least partly dependent on the context in which the action decision is made.

Furthermore, it has been previously shown that activation in frontoparietal cortex is strongly task-dependent [e.g. 60] [but see 61] and that task information also differs across different sequential stages of a task [[62], [63], [64], [65], [66]]. Our results extend these findings by showing that task representations can be relatively invariant to certain changes (i.e., variation in reason) but not to others (i.e., changes in context).

It has been shown behaviourally that context affects action responses [67], and intuitively it seems necessary that action intentions have context-dependent elements, as otherwise people would not be able to consider contextual factors in establishing appropriate actions. Yet, our results point towards a stronger form of context-dependency. Decoding accuracy was not only significantly lower when testing across context (compared to within context), for most brain regions and conditions there was moderate evidence that the intention-related information did not generalize across different contexts. There are two possible explanations for this result: 1) There are nevertheless common representational cores underlying intentions for the same action across contexts, but this core was not accessible via the sampling of the neural signal with fMRI (i.e., a false negative result); or 2) there is no context-invariant representational core at all. We will discuss both these options.

Our findings could reflect a false negative: Hypothetically, when parts of the processes change with context and parts remain invariant, the changing parts may obscure the invariant representations, and decrease the signal-to-noise ratio, which will make decoding harder. Yet, our Bayesian analyses suggests that this may not be the case, since they reveal anecdotal to moderate evidence that neural intention representations are not context-invariant. More complex coding schemes are conceivable in which the context representation is convolved with the intention representation in a non-linear way [68]. The non-linear convolution defies detection with our classification paradigm.

Alternatively, our findings could be interpreted as pointing to a different possibility: Intentions do not have a context-invariant representational core at all. The negative Bayes factors provided anecdotal to moderate evidence in favour of this hypothesis. In that case, action-control processes could potentially be understood as a dynamic integration of sensorimotor processes tailored to the relevant context [55,69], with an action that is uniquely adapted to the context, rather than the formation of an invariant intention that is subsequently translated into the current context [20,70,71]. This interpretation raises multiple fundamental research questions. What are the reported brain areas doing during intentional action if not constituting a context-free representation of an action (see Uithol et al., 2014)? How is generalisation between contexts possible if the activity in much of the neural circuitry involved in intentional action is context-dependent?

Despite our efforts to improve ecological validity of our paradigm by varying contexts and reasons, there were still aspects of our design that make it different from everyday intention formation: 1) the context was not really “immersive” and instead participants had to imagine the context by means of a photograph on the screen; and 2) participants did not execute the exact action they had chosen, but they indicated their choice via a button-press, thus requiring an additional transformation and level of abstraction.

In our design there was a 6 s delay between the onset of the intention formation and the moment the participant could indicate the chosen option. This was introduced as in other studies in order to maximize the separation of the relevant stage for the multivariate analyses [see e.g. 9]. In order to avoid making the design more complex, we did not ask participants for additional introspective reports during this delay. However, we have no indication that they were doing anything other than maintaining the chosen option in mind (as reflected in the high accuracy rate of the responses). Participants were asked to imagine themselves in the indicated context and to imagine the chosen action.

Our results, stemming from a task in which meaningful actions in specific contexts were selected (even though only being indicated through a button press), can be compared to previous fMRI studies in which actions more complex than a button press were selected and executed, but in a meaningless context [57,[72], [73], [74], [75]]. Our study still employs a simplification compared to real-world intentions, insofar as the action context are only imagined and that actions are simplified to mere button presses, the latter due to limitations in moving inside the scanner bore. Previous studies employed other simplifications, in the sense that typically actions lacked an ecological purpose (i.e., an ecologically valid reason) and a meaningful context. Nevertheless, there is some overlap in the cortical areas from which information could be decoded, including ventral premotor, lateral prefrontal and parietal areas, primarily inferior parietal lobule, superior parietal lobule, intraparietal sulcus and precuneus. On the other hand, many of the regions that have been previously reported for being involved in action selection are absent in our results: frontal eye fields, dorsal premotor and –of course– primary motor areas, frontopolar and medial prefrontal cortex, as well as pre-supplementary motor area [6,10,42,60,73,76]. In previous studies, these regions were often mentioned to be involved in intentional action [77], yet, we found no information in these areas in our study.

It remains to be seen, though, whether the differences between our findings and previously reported findings are robust and replicable [78], but we can think of two explanations that could account for this potential discrepancy in the implicated brain regions: 1) the same areas are actually involved but the activity pattern is not (significantly) different between the two intentions we manipulated in our study; or 2) since our study did not involve executing the selected action (the button press only related to the chosen action in an arbitrary way), no action-planning component specific to the chosen action is involved, which may be necessary for these additional areas to be recruited. Previous studies that dissociated decision processes from overt movement [42,[79], [80], [81]] did find intention-related information in medial PFC. This could suggest that the neural loci of intention-related activation are dependent on the nature of the decision and execution, which would make generalising across paradigms difficult [69].

What the exact relation between context and intention representation is, is still not clear. Is, for instance contextual similarity correlated with intention decodability? Do various contexts cluster together in a functional way, rather than a purely visual way, when the task is forming intentions? And if so, would this clustering also find its way into occipital regions, as this task-driven visual processing is suggested in a recent study on conceptual categorisation [82]?

Next to empirical questions, conceptual work is needed to advance our understanding of intentional action. The borders between action planning, action decision, and action understanding may be clear from a conceptual point of view, the overlap in contributing brain regions (e.g. Molenbergh et al. (2012) found context modulation in ventral premotor cortex and inferior parietal lobule during action observation) suggest that from a neural point of view these processes may be related.

To conclude, multivariate methods allow tracing stable states in action decision processes. Our results show that the intuitive conclusion that these states are context-independent representations of action decisions, akin to the context-free nature of the notion of “intention” in everyday language and folk psychology, is potentially problematic. States underlying an intention that seem stable within a given research paradigm, may only be stable within a certain range of factors. In our experiment, the stable character of intentional processes did not generalize to different contexts.

## Author contribution statement

Sebo Uithol: Conceived and designed the experiments; Performed the experiments; Analyzed and interpreted the data; Wrote the paper.

Kai Görgen: Conceived and designed the experiments; Performed the experiments; Analyzed and interpreted the data; Contributed reagents, materials, analysis tools or data; Wrote the paper.

Doris Pischedda: Performed the experiments; Analyzed and interpreted the data; Wrote the paper.

Ivan Toni: Analyzed and interpreted the data; Wrote the paper.

John-Dylan Haynes: Conceived and designed the experiments; Analyzed and interpreted the data; Contributed reagents, materials, analysis tools or data; Wrote the paper.

## Data availability statement

Data will be made available on request.

## Declaration of competing interest

The authors declare that they have no known competing financial interests or personal relationships that could have appeared to influence the work reported in this paper.

## Footnotes

1 Goals (and therefore intentions) can be defined at many different levels [19,36]. Consequently, actions that are purposeless on one level (say the movement in a Libet-like experiment) can be attributed to a goal at a higher level (e.g., completing the experiment or complying with the experimenter).
