# Supplementary material for: The effect of context and reason on the neural correlates of intentions
Source: Heliyon. 2023 Jun 15;9(6):e17231. doi: 10.1016/j.heliyon.2023.e17231 (PMC10293734; doi:10.1016/j.heliyon.2023.e17231)
Supplement: Multimedia component 1 [file mmc1.docx]

**Supplemental Material**

Overview trials

| **Context** | Breakfast | | | | Supermarket | | | |
| --- | --- | --- | --- | --- | --- | --- | --- | --- |
| **Intention** | Open | | Place | | Open | | Place | |
| **Reason** | Drink | Smell | Keep | Put away | Drink | Smell | Keep | Put away |
| **Description** | 1 | 2 | 3 | 4 | 5 | 6 | 7 | 8 |

1. You want to drink some milk so you have grasped the milk box.
2. You are not sure whether the milk is still good, so you decide to smell it.
3. You have filled your glass with milk, and you have put the lid back on.
4. You have emptied the bottle in your glass, and no longer need it.
5. You are really thirsty. You have the money so you decide that it is ok to start drinking before paying.
6. The last box of milk is past the best before date, so you decide to smell it first.
7. You picked the milk that you want to buy from the shelf to put in the cart.
8. You picked up the milk, but you realize that you already have enough milk at home, so you do not need it.

(These are the English translations of the originally sentences in German)


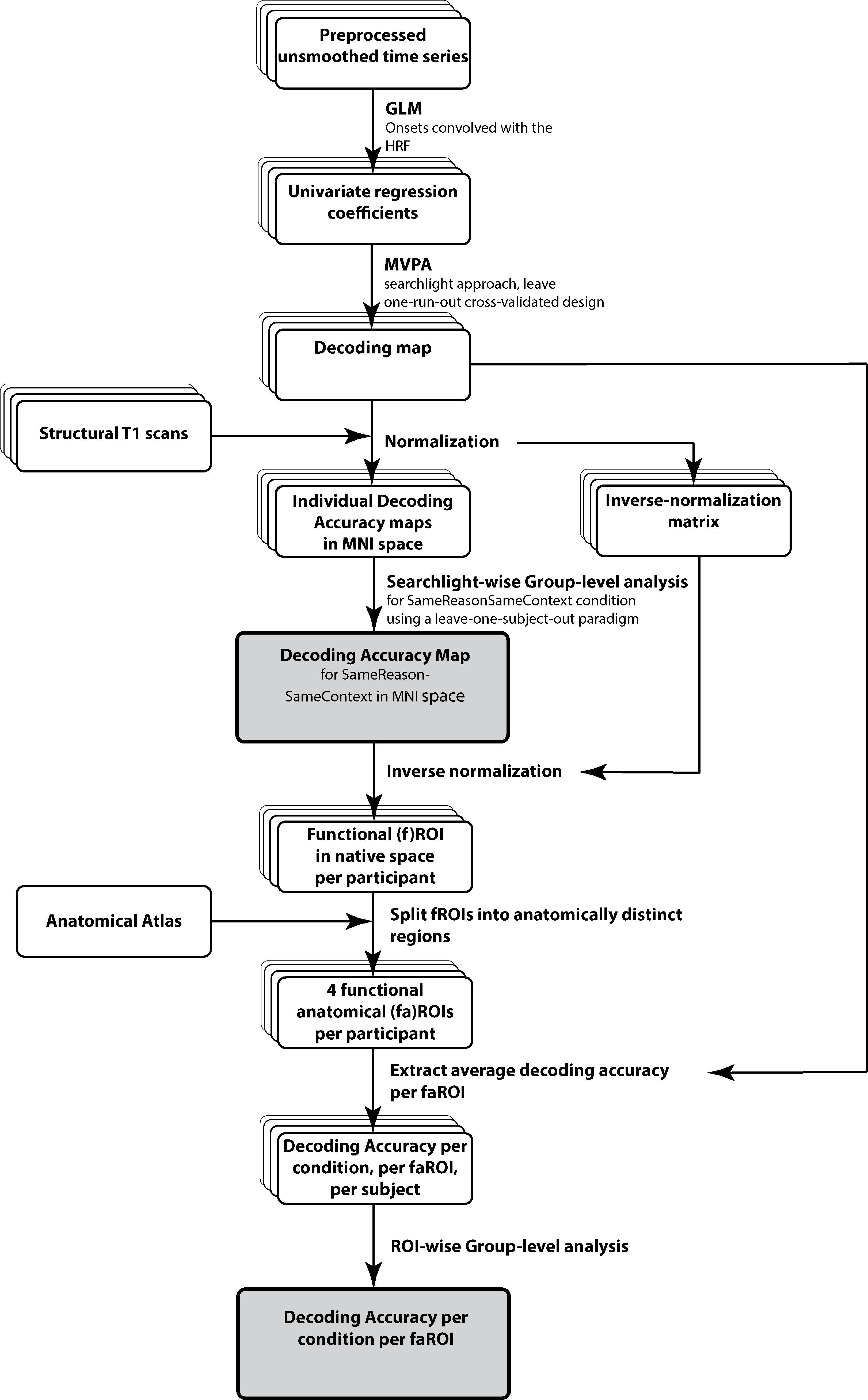


**Figure S1**. Schematic overview of the analysis pipeline.

|  | **X** | **Y** | **Z** |  |
| --- | --- | --- | --- | --- |
| **SameReason-SameContext** | -60 | -60 | 10 | Left Angular Gyrus |
|  | -52 | 4 | 42 | Left Premotor Cortex |
|  | 66 | -14 | -6 | Left Operculum |
|  | 16 | -78 | -25 | Cerebellum |
|  |  |  |  |  |
| **CrossReason** | 14 | -58 | 30 | Right dlPFC |
|  | -8 | 2 | 50 | left PM/preSMA |
|  | 12 | -50 | -22 | Cerebellum |
|  | -50 | -58 | 12 | left Angular Gyrus |
|  | -50 | 24 | 18 | left Pars Triangularum |
|  | -24 | 48 | 28 | left anterior PFC |
|  |  |  |  |  |
|  |  |  |  |  |
|  |  |  |  |  |
| **CrossContext** | -52 | -62 | 12 | left Angular Gyrus |
|  |  |  |  |  |
| **CrossReason-crossContext** | -50 | -56 | 6 | left Angular Gyrus |
|  | -8 | -70 | 40 | Superior Parietal Lobule |
|  | -16 | 44 | 46 | left Frontal Eyefields |

**Table S1: Coordinates of peak decoding accuracy per condition**
